# Bronchial Artery Embolization in Life-Threatening Hemoptysis: Outcome and Predictive Factors

**DOI:** 10.5334/jbsr.2310

**Published:** 2021-02-01

**Authors:** Kinley Dorji, Keerati Hongsakul, Warissara Jutidamrongphan, Maliwan Oofuvong, Sarayut Geater

**Affiliations:** 1 Prince of Songkla University, TH

**Keywords:** bronchial artery, embolization, clinical success, recurrence hemoptysis, angiography

## Abstract

**Purpose:**

To determine the outcome and predictive factors of clinical success of bronchial artery embolization in life-threatening hemoptysis.

**Material and Methods:**

We reviewed all bronchial artery embolization procedures performed for life-threatening hemoptysis between January 2008 and December 2018. The outcomes and predictive factors of clinical success following embolization were evaluated.

**Results:**

One hundred and eighty-four bronchial artery embolization procedures performed in 145 patients were eligible for the study. The technical and clinical success rates of the procedures were 170/184 (92.4%) and 129/184 (70.1%), respectively. The unstable hemodynamics and prothrombin time/international normalized ratio >1.5 was associated with lower clinical success rate, while embolization of more than one vessel was associated with higher clinical success rate.

**Conclusion:**

Bronchial artery embolization is a safe and effective procedure for controlling bleeding in life-threatening hemoptysis. However, low clinical success rate was noted in patients with unstable hemodynamics and coagulopathy, while multiple vessel embolization was associated with higher clinical success.

## INTRODUCTION

Hemoptysis is defined as expectoration of blood originating from the tracheobronchial tree or pulmonary parenchyma. Although hemoptysis is not dangerous, about 5–15% can be life-threatening, with mortality rate more than 50% if managed inappropriately [1].

Since its introduction by Remy et al. [2] as an alternative to surgery, bronchial artery embolization (BAE) has established itself as the first-line treatment of life-threatening hemoptysis. It is a minimally invasive endovascular procedure with lower morbidity and mortality as compared with surgery. Several studies have validated the efficacy and safety of BAE. It has a high technical and clinical success rate with an acceptable major complication rate [3,4]. However, one of the main drawbacks of BAE is its high recurrence rate [5]. Although a handful of studies evaluated the predictive factors of recurrent hemoptysis, no study evaluated the predictive factors of clinical success rate.

This study was undertaken to determine the outcomes and predictive factors influencing the clinical success following BAE in life-threatening hemoptysis.

## MATERIALS AND METHODS

### PATIENTS

This historical cohort study was approved by an institutional review board. A total of 289 BAEs were performed in 260 patients, from 2008 to 2018. The inclusion criteria were patients who presented with life-threatening hemoptysis and more than 15 years old. The exclusion criteria were patients who had hemoptysis caused by pulmonary circulation or prophylactic and/or elective BAE. Finally, a total of 184 BAEs from 145 patients were enrolled.

Patients’ demographic and clinical data, including laboratory parameters, were obtained from the hospital information system. Angiographic image and BAE procedure details were retrieved from picture archiving and communication system and radiology information system.

### PROCEDURE

BAE was performed through standard percutaneous transfemoral catheterization using 5-French (Fr) vascular sheath under fluoroscopic monitoring and local anesthesia. A 5-Fr diagnostic catheter using a Mikaelsson catheter (Boston Scientific Corporation, MA, USA) was introduced following the right or left common femoral artery. Bronchial arteries were selected corresponding to the side of lung pathology, and selective hand-injection angiography was then performed to identify the source of bleeding. If initial selective catheterization of bronchial artery was not successful, flush aortogram was done using 5-Fr pigtail catheter (Boston Scientific Corporation, MA, USA) to help identify the anatomy and origin of bronchial arteries. Embolization was performed once the bleeding artery was selected and if the catheter was stable. If the 5-Fr catheter was not stable, or if we were unsure of the anastomosis to the anterior spinal artery, superselection using 2.8-Fr microcatheter (Renegade Hi-Flo; Boston Scientific Corporation, MA, USA) was performed followed by angiography with hand injection of nonionic contrast media. For upper lobe disease, especially in recurrent hemoptysis, subclavian artery angiography was also done to identify possible coexistent non-bronchial systemic collaterals. Similarly, for lower lobe disease, inferior phrenic artery was also evaluated.

The embolic agents used were 355–500 µm of polyvinyl alcohol (PVA) (Contour; Boston Scientific Corporation, NY, USA) and gelatin sponge (Spongostan; Ethicon, NJ, USA). In some cases, a combination of PVA and gelatin sponge was used. A post-embolization angiography was performed to confirm technical success. After the procedure was finished, the vascular sheath was removed, and manual compression was performed for hemostasis.

### DEFINITIONS

Outcomes were defined following the Society of Interventional Radiology, Standards of Practice Committee [6]. The technical success was defined as the ability to catheterize and embolize the abnormal vessel as confirmed by angiography. Clinical success was defined as either complete cessation of hemoptysis or significant reduction in hemoptysis, resulting in clinical improvement without requiring further intervention within the same admission and/or within 30 days after BAE. Major complication was defined as sequelae, which were permanent or a cause of death, requiring treatment or prolonged hospitalization. Minor complication was complication that did not require treatment and was self-limited.

### STATISTICAL ANALYSIS

The categorical variables were presented as descriptive statistics. The continuous variables were divided into categories and were also presented as descriptive statistics. The association between the predictive factors and the clinical success was analyzed using multivariable logistic regression analysis. A *p* value <0.05 was considered to be statistically significant.

## RESULTS

A total of 145 patients with 184 BAEs were eligible for this study. ***Table 1*** summarizes the demographic and clinical parameters of the patients. Of the 145 patients, 66.2% were male, and 33.8% were female with mean age of 63.9 ± 16.3 years. Most of enrolled patients (79.3%) were hemodynamically stable at time of admission. One hundred patients (68.9%) had a past history of hemoptysis. Only 14 patients had previous BAE procedure. The most common cause of hemoptysis were post-TB sequelae (51.0%). Meanwhile, in the category of others, which constituted 13 patients, the most frequent cause was necrotizing pneumonia.

**Table 1:** Demographic data and clinical variables (n = 145).


VARIABLES	NUMBER (%)

**Sex**	
Male Female	96 (66.2) 49 (33.8)

**Age**	
(years) mean ± SD	63.9 ± 16.3

**Hemodynamics**	
Stable (≥90/60 mmHg) Unstable (<90/60 mmHg or on inotropic drug)	115 (79.3) 30 (20.7)

**Past history of hemoptysis**	
Yes No	100 (68.9) 45 (31.1)

**Past history of embolization**	
Yes No	14 (9.7) 131 (90.3)

**Co-morbidities:**	
Hypertension Diabetes mellitus Dyslipidemia Chronic kidney disease Chronic liver disease Others	41 (28.3) 17 (11.7) 17 (11.7) 4 (2.8) 3 (2.1) 48 (33.1)

**Pre-BAE hemoglobin** (g/dL)	
< 8 8–10 > 10	21 (14.5) 50 (34.5) 74 (51.0)

**Platelet count** (×10^3^/µL)	
<70 70–150 >150	4 (2.8) 10 (6.9) 131 (90.3)

**PT/INR**	
≤1.5 >1.5	141(97.2) 4 (2.8)

**Hematocrit** (%)	
<25 25–30 >30	16 (11.0) 46 (31.7) 83(57.3)

**Etiology**	
Post-Tuberculosis Bronchiectasis Aspergillosis Active tuberculosis Malignancies Idiopathic Others	74 (51.0) 31 (21.4) 29 (20.0) 20 (13.8) 17 (11.7) 7 (4.8) 13 (9.0)


PT/INR, prothrombin time/international normalized ratio.

**Figure 1:**
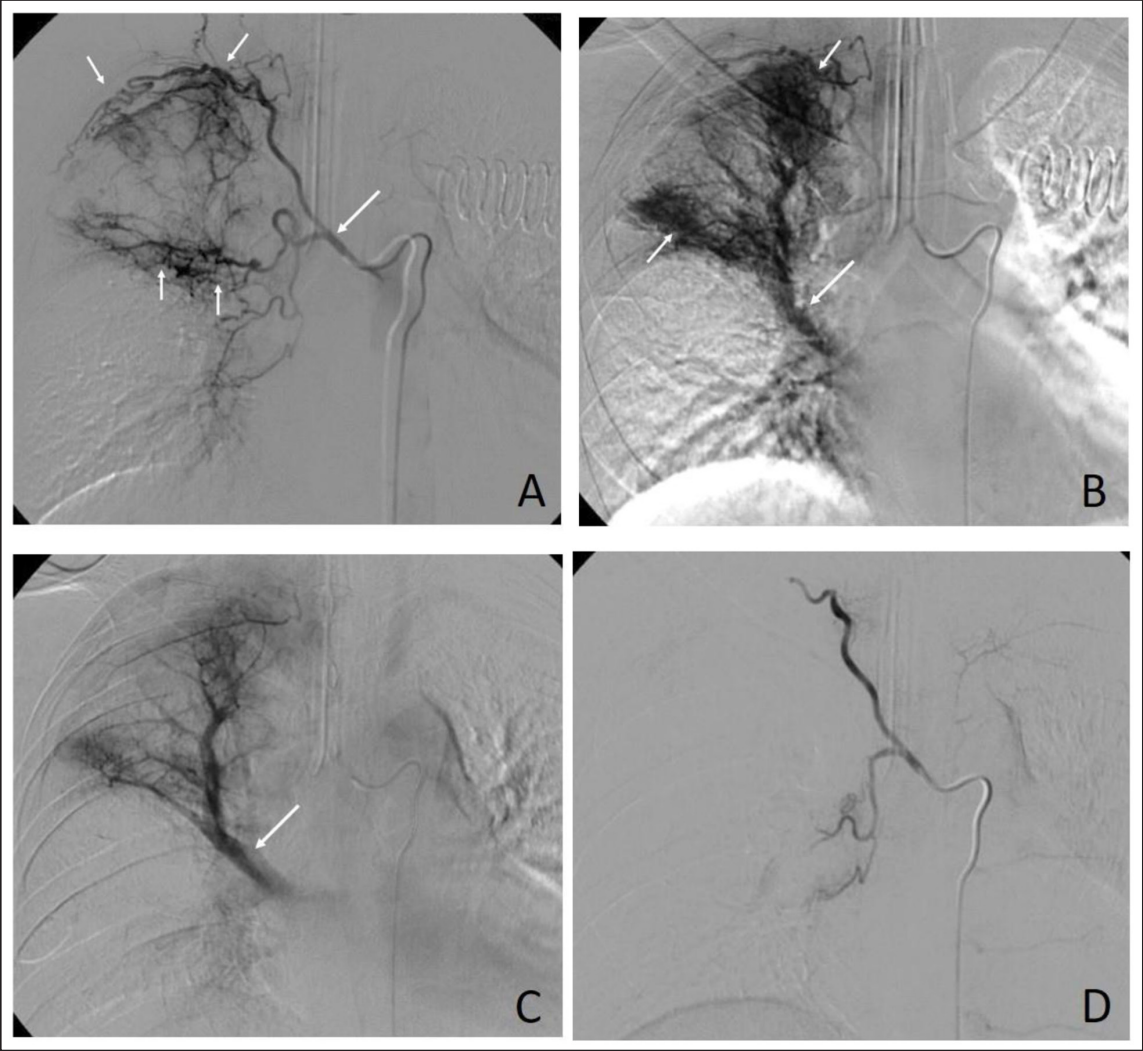
A 54-year-old male patient with a history of old pulmonary tuberculosis. **(A)** Selective right intercostobronchial angiography showing hypervascularity of lung parenchyma at the right upper lobe (small arrows) feeding from the dilated right intercostobronchial artery (large arrow). **(B)** Parenchymal and **(C)** delayed phases of angiographies showing obvious hypervascularity of lung parenchyma (small arrows in B) and shunting draining to the upper lobe branch of the right pulmonary vein (large arrows in B and C). **(D)** An angiogram after embolization with polyvinyl alcohol showing complete occlusion of right intercostal and bronchial arteries.

***Table 2*** presents the detail of angiographies and embolizations. The most commonly encountered abnormalities on angiographies were hypervascularity of lung parenchyma (95.7%) (***Figure 1***). Pseudoaneurysm was found in 3.3%, and contrast extravasation was seen only in 1.1%. Technical success rate was found in 170/184 (92.4%) procedures. The PVA was the most common used embolic agent (58.7%). Clinical success was achieved in 129 procedures (70.1%). Recurrent hemoptysis was found in almost half of the procedures. The overall complication rate was 6.0%. Almost all of them were minor complications. The major complication was seen in one case, in which the patient presented with bilateral lower limb paraplegia and paresthesia, which likely to have occurred due to anterior cord ischemia. There was no BAE-related death in this study.

**Table 2:** Detail of angiography and embolization (n = 184).


VARIABLES	NUMBER (%)

**Angiographic findings**	
Hypervascularity of lung parenchyma Hypertrophy of bronchial artery Bronchopulmonary shunt Non-bronchial systemic collaterals Pseudoaneurysm Contrast extravasation Multiple feeders	176 (95.7) 168 (91.3) 77 (41.8) 101 (54.9) 6 (3.3) 2 (1.1) 117 (63.6)

**Embolic agents**	
PVA Gelatin sponge Combination (PVA + gelatin sponge)	108 (58.7) 36 (19.6) 40 (21.7)

**Number of vessels embolized**	
1 ≥2	65 (35.3) 119 (64.7)

**Outcome**	
Technical success	170 (92.4)
Clinical success	129 (70.1)
Recurrence	90 (48.9)
Complications	
No complications Minor complications Major complications	173 (94.0) 10 (5.4) 1 (0.6)


***Table 3*** presents multivariable logistic regression analysis for the clinical success. Patients with unstable hemodynamics and prolonged prothrombin time (PT)/international normalized ratio (INR) >1.5 are associated with lower chances of having clinical success. On the other hand, embolization of more than one vessel had 9.8 times higher chance of having clinical success.

**Table 3:** Multivariate logistic regression analysis for clinical success.


VARIABLES	CRUDE ODD RATIOS (95% CI)	ADJUSTED ODD RATIOS (95% CI)	*p*-VALUE

**Age group**			
≤ 30 31–50 51–80 > 80	1.000 1.875 (0.497–7.079) 2.069 (0.579–7.392) 2.500 (0.484–12.925)	1.000 8.365 (0.632–110.799) 5.645 (0.367–86.750) 1.878 (0.115–30.531)	. 0.107 0.214 0.658

**Gender**			
Male Female	1.000 1.219 (0.598–2.483)	1.000 2.752 (0.937–8.083)	1.000 0.065

**Hemodynamic status**			
Stable Unstable	1.000 0.529 (0.249–1.121)	1.000 0.288 (0.106–0.786)	. 0.015*

**Past history of hemoptysis**	0.505 (0.216–1.181)	0.452 (0.168–1.220)	0.117

**Past history of BAE**	0.980 (0.469–2.047)	2.072 (0.640–6.714)	0.224

**Co-morbidities**			
Hypertension Diabetes mellitus Others	2.381 (1.124–5.044) 3.487 (0.765–15.902) 1.791 (0.886–3.618)	1.895 (0.719–4.997) 3.337 (0.384–28.993) 1.569 (0.481–5.115)	0.196 0.275 0.455

**Etiologies**			
Active tuberculosis Post-tuberculosis Aspergillosis Bronchiectasis Malignancy	1.041 (0.450–2.409) 0.767 (0.398–1.476) 0.442 (0.221–0.881) 2.694 (0.946–7.674) 0.548 (0.203–1.478)	2.237 (0.408–12.265) 2.122 (0.655–6.870) 0.388 (0.140–1.075) 5.153 (0.860–30.864) 0.401 (0.091–1.768)	0.354 0.209 0.069 0.073 0.228

**PT/INR**			
≤1.5 >1.5	1.000 0.100 (0.052–0.010)	1.000 0.091 (0.011–0.757)	. 0.027*

**Number of vessel embolized**			
1 vessel ≥2 vessels	1.000 1.489 (0.758–2.924)	1.000 9.840 (1.433–67.590)	. 0.020*

**Angiographic findings**			
Hypertrophic feed Hypervascularity BP shunt NBSC Pseudoaneurysm Multiple feeders	0.765 (0.238–2.456) 1.293 (0.247–6.777) 0.810 (0.424–1.549) 1.132 (0.589–2.177) 0.848 (0.150–4.804) 1.114 (0.570–2.179)	0.389 (0.081–1.858) 1.957 (0.141–27.201) 0.701 (0.242–2.026) 1.741 (0.255–11.905) 1.418 (0.246–8.173) 0.401 (0.067–2.381)	0.236 0.617 0.511 0.572 0.696 0.314

**Embolic agents**			
Gelatin sponge PVA Combination	1.000 0.828 (0.363–1.887) 0.556 (0.214–1.445)	1.000 0.946 (0.304–2.941) 0.538 (0.143–2.027)	. 0.923 0.359


BP-bronchopulmonary, NBSC-Non-bronchial systemic collaterals, * *p* < 0.05.

## DISCUSSION

The most common cause of hemoptysis in our study was post-tuberculosis sequelae. This is in line with other studies conducted in Asia and developing world which revealed tuberculosis and post-tuberculosis sequelae as the most common etiology for hemoptysis [7]. In contrast, cystic fibrosis and sarcoidosis are more common in Western countries [7]. In our study, the common angiographic finding was lung parenchymal hypervascularity which were similarly reported in previous publications [1,7]. The other less common but significant angiographic findings included bronchopulmonary shunt and the presence of non-bronchial systemic collaterals. Consistent with other studies, pseudoaneurysm and contrast extravasation were not commonly observed in this study. However, Syha et al. [8] reported relatively higher findings of contrast extravasation (16%) in their study.

The technical success rate in this study was high, which was compatible with most studies, ranging from 90% to 100% [3,9]. However, clinical success was achieved in 70.1% of the cases, which is relatively low compared to most of the studies, which range from 82% to 100% [9,10]. However, a study by van den Huevel et al. [5] reported a quite similar clinical success rate of 67%. The main reason for this relatively low clinical success rate could be due to differences in the definition of the clinical success. In most of the abovementioned studies, they assessed the immediate clinical success rate, which was defined as bleeding control within 24 hours after BAE. In our study, the clinical success extended beyond 24 hours until the patient was discharged, during which they did not require any further interventions. Our study has a recurrence rate of 48.9%, which was quite high and consistent with most studies after 2010, with recurrence rates ranging from 9.8% to 57.5% [5,9,11]. This high range of recurrence rate was related with long-term patient follow-up [12]. Studies having short duration follow-up are likely to have low recurrence rate as compared to those studies that have been followed up for a longer duration. Our study was one of the many studies with long-term follow-up data; therefore, the recurrent rate of hemoptysis was quite high.

With advances in technology and technique, the complication of BAE is gradually diminishing over the years making it safe. In our study, 5.4% of the cases had minor complications, which consisted mostly of post-embolization syndrome and groin hematoma. Anterior spinal cord infarction is one of the feared complications of BAE with a reported incidence between 1.4% and 6.5% [13,14]. This study had one case of anterior spinal cord infarction. After retrospective review, we found that we missed an anterior spinal artery from the initial bronchial angiography; therefore, nonselective embolization was performed, and the patient finally developed persistent paraplegia. To avoid this major complication, carefully reviewed pre-procedural angiogram and superselective embolization should be performed. Other major complications such as transverse myelitis, bronchial infarction, transient cortical blindness, and stroke were not observed in this study [12].

Because BAE has a good clinical success, it is considered as the first-line treatment in managing life-threatening hemoptysis. There was no literature evaluating the predictive factors of clinical success. Our study found three predictive factors influencing the clinical success including hemodynamic status, PT/INR, and the number of embolized vessels. The unstable hemodynamic status was defined as blood pressure <90/60 mmHg or on inotropes to maintain normal blood pressure. The study by Mossi et al. [15] observed that altered hemodynamics was associated with increased risk of recurrence although they did not evaluate the association with clinical success. PT is the time taken for the blood to clot. Instead of presenting it as a time unit, it is expressed as a ratio, which is called INR. A PT/INR of more than 1.5 is considered as prolonged coagulation. Our result showed that prolonged coagulation has less likelihood of clinical success. This makes sense, as the entire process of BAE is to promote clotting by making the flow stagnant. This result would imply that we must first correct the coagulopathy before performing embolization to achieve greater success. However, our result was contrary to the results by Chun et al. [16], which shows no significant relationship between clinical response and coagulation. The embolization of ≥2 vessels was found to be associated with greater chances of having clinical success. Having more embolized vessels means a high chance of stopping the bleeding, particularly in the case of destroyed lung as post-TB sequelae with multiple arterial supply from brachial and non-bronchial systems.

The strength of the study is in the determination of predictive factors of clinical success, which was not evaluated in previous studies. However, one limitation to our study must be noted; it is a single-centered study.

BAE is a safe and effective procedure in the management of life-threatening hemoptysis. However, lower clinical success rate was observed in patients with unstable hemodynamics and prolonged coagulation, while multiple vessel embolization was associated with higher clinical success.
